# Endodontic Considerations in Three-canalled Premolars: A Practical Update

**DOI:** 10.7508/iej.2016.02.012

**Published:** 2016-03-20

**Authors:** Zahed Mohammadi, Sousan Shalavi, Luciano Giardino, Saeed Asgary

**Affiliations:** a *Iranian Center for Endodontic Research, Research Institute of Dental Sciences, Dental School, Shahid Beheshti University of Medical Sciences, Tehran, Iran; *; b * Iranian National Elites Foundation, Tehran, Iran;*; c * Private Practitioner, Hamedan, Iran; *; d *Department of Periodontology, Endodontology, Pharmacology and Microbiology, Dental School, University of Brescia, Italy*

**Keywords:** Mandibular Premolars, Maxillary Premolars, Mini-Molars, Root Canal Anatomy, Three-Canalled Premolars

## Abstract

The most difficult clinical considertions in orthograde root canal treatment are generally related to the anatomy of the teeth. Three-canalled maxillary and mandibular premolars (mini-molars) have been reported in several studies. The purpose of this paper was to review various aspects of three-canalled premolars including incidence, clinical and radiographic diagnosis, racial predisposition, access cavity preparation, instrumentation and obturation.

## Introduction

Consistent, high levels of success in endodontic treatment require an understanding of root canal anatomy and morphology. To achieve endodontic success, the entire root canal system must be debrided, disinfected and obturated. The clinician must have a thorough understanding of normal anatomy common variations from the norm. The clinician must also be prepared to identify those teeth that tend to vary greatly from the norm, *e.g*. mandibular premolars [1].

## Materials and Methods

***Retrieval of Literature***

A Medline search was performed through the English articles published from 2000 to 2014. The searched keywords included “Three-Rooted Premolar”, “Three-Canalled Premolar”, “Three Canals AND Premolars”, “Three Canals AND Maxillary Premolars", "Three Canals AND Mandibular Premolars" and "Radiculous". Then, a hand search was done in the references of result articles to find more matching papers.

## Results

A total of 470 articles were found which in descending order of their related keywords are “Three-Rooted Premolar (34)”, “Three-Canalled Premolar (2)”, “Three Canals AND Premolars (241)”, “Three Canals AND Maxillary Premolars (63)", "Three Canals AND Mandibular Premolars (125)" and "Radiculous (5)".

## Discussion

***Incidence in*** ***maxillary*** ***premolars***

The root canal anatomy of three rooted maxillary premolars is very similar to that of maxillary molars. Therefore, the terms mini-molars and radiculous have been used to describe them [2, 3]. 

Maxillary first premolars have two root canals in 50-90% of cases. However, one and three root canals have also been reported. In 10-50% of cases, one root canal has been reported [4]. 

Maxillary first premolars may possesses three root canals as well (mesiobuccal, distobuccal and palatal), the incidence of which varies from 0 to 6% [3, 5, 6] (Tables 1 and 2). 

For second premolar, laboratory studies have demonstrated a lower incidence of three root canals which is between 0.3 and 2% [7-9]. A study showed that only 1.1% of teeth with three canals and did not report any with three roots [2]. 

The possibility of three roots in maxillary second premolars is quite small and only few cases have been reported. Vertucci [8] showed that the incidence of three canals in maxillary first and second premolars were 5% and 1%, respectively. According to Pineda and Kuttler [10] the incidence of three canals in maxillary first and second premolars were 0.5% and 0%, respectively. It has been revealed that the incidence of three canals in maxillary first and second premolars were 1.7% and 0.7%, respectively [5]. Pecora *et al.* [9] revealed that the incidence of three canals in maxillary first and second premolars were 2.5% and 0.3%, respectively (Tables1 and 2). 

***Incidence in mandibular premolars***

Mandibular premolars can be the most difficult teeth for endodontic treatment. This fact is due to the presence of multiple root canals, apical deltas and lateral canals [11]. Several studies have assessed the root canal morphology of mandibular premolars over the years [12-15]. Mandibular first premolars usually have one canal (67-86%). However, in 15-25% of cases two canals are present. Three canals have been reported in mandibular first premolars in 0-33% of cases [12, 13, 15]. The incidence of two and three canals in mandibular second premolars are less than mandibular first premolars. The incidence of three canals in mandibular second premolars is 0-8% [12-14]. According to Rahimi *et al.* [16], the incidence of three canals in mandibular first and second premolars were 1.2% and 0%, respectively (Tables 3 and 4). 

Vertucci [17] reported the incidence of three canals in mandibular first and second premolars in 0.5 and 0% of cases, respectively. Pineda and Kuttler [10] reported three canals in mandibular first and second premolars in 0.9% and 0%, respectively. Caliskan *et al.* [18] showed that the incidence of three-canalled mandibular first and second premolars were 5.7% and 0%, respectively. Zillich and Dowson [19] showed that the incidence of both three-canalled mandibular first and second premolars were 0.4% (Tables 3 and 4). 

***Diagnosis***

***a) Clinical Examination***

A careful clinical examination can furnish the clinician with signs that are typical of the presence of a third root. In the buccal aspect, the gingiva has a flat appearance and its convexity is not in harmony with the adjacent elements. Probing can disclose the presence of a buccal furcation; sometimes a small depression can be present, starting from the buccal roots furcation and proceeding in a coronal direction for a few millimeters, remaining mainly localized in the cervical third of the tooth crown [20, 21]. 

**Table 1:** Incidence of three roots in the maxillary first premolars

**Author **	**Year**	**Incidence (%)**
**Ingle **	1965	2.0
**Carns and Skidmore [** 3 **]**	1973	6.0
**Vertucci and Gegauff [** 5 **]**	1978	4.0
**Pecora ** ***et al.*** ** [** 22 **]**	1991	2.5
**Loh ** ***et al*** **. [** 6 **]**	1996	0.0
**Kartal ** ***et al.*** ** [** 5 **]**	1998	1.3
**Chaparro ** ***et al*** **. **	1999	3.3

Gingival recession may reflect the furcal morphology in these teeth and thus hint at the presence of two buccal roots.

Probing the buccal sulcus to feel the root eminences and furcal anatomy may also help to identify the presence of two buccal roots if present [23].

The use of magnification and fiber optic illumination offers a tremendous advantage in locating and treating *extra* canals [24]. The surgical operating microscope has been found to be particularly helpful.

Examination of the pulp chamber is one of the diagnostic measures. It is beneficial to use 17% aqueous EDTA, 95% ethanol and the Stropko irrigator with a 27-gauge notched endodontic irrigating needle to clean and dry the pulp chamber prior to visually inspecting the canal system [25].

***b) Radiographic Examination***

Accurate preoperative radiographs, straight and angled, using parallel technique are essential in providing clues as to the number of roots that exist [26].

In mandibular premolars with three canals, the cervical half of the root is generally wider than usual, with little or no taper. Root canals may not be evident radiographically or may look unusual. Root canal space may disappear halfway through the roots. Careful interpretation of the periodontal ligament space may suggest the presence of extra root/canal(s). Mesial and distal angled views will often reveal the presence of a bi/trifurcation of the root canal. Clinical presentation is frequently atypical [23]. 

The limit of a radiographic examination is the production of a two-dimensional image of a three dimensional root canal system; to achieve a better evaluation of the three-dimensional structure of the tooth, three preoperative radiographs often have to be taken at different angles [26]. Nevertheless, a good quality parallel radiography can provide enough evidence of internal and external anatomy of the root to suggest the presence of a third canal. By analyzing the external anatomical details, the presence of a third root canal can be suspected whenever the radicular anatomy is not clearly visible or distinct. If the buccal roots are separated, it is sometimes possible to distinguish the furcation cortical bone [23, 26].

Another external aspect of anatomy to be considered is that a premolar tooth with three roots has a small taper; when the mesiodistal diameter of the middle third of the root is equal or greater than the mesiodistal diameter of the crown, a third root canal is likely to be present [23, 26]. Considering the internal root anatomy, the analysis has to be conducted following the run of the canal proceeding from the pulp chamber toward the apex. If the root canal suddenly seems to broaden and straighten, or if it losesits radiolucency such that its course cannot be followed anymore, it should be suspected that the presence of a second canal in the same root or a canal in another root superposed on the first one is likely [27, 28]. Careful interpretation of the periodontal ligament space may suggest the presence of an extra root or canal. Mesial and distal angled views will often reveal the presence of a bi/trifurcation of the root canal [28, 29].

**Table 2:** Incidence (%) of three canals in maxillary first premolars

**Author**	**Year**	**Incidence (%)**
**Pineda and Kuttler [** 10 **]**	1972	0.5
**Green **	1973	0.0
**Carns and Skidmore [** 3 **]**	1973	6.0
**Pecora ** ***et al.*** ** [** 9 **]**	1991	2.5
**Kartal ** ***et al.*** ** [** 5 **]**	1998	1.7

CBCT is also very helpful and provides comprehensive information about the root canal morphology of maxillary and mandibular premolars. These data may help clinicians in root canal treatment of premolar teeth [30].

***Racial predisposition***

There seems to be a racial predisposition for the presence of two or more canals in maxillary and mandibular premolars [13, 31].

The ethnic background of the patients with three rooted maxillary premolars in many of the studies was not identified. The studies identifying ethnic background have demonstrated distinct differences between Asian and Caucasian populations. Single rooted maxillary first premolars are the dominant form in Asian population [22, 32] and three rooted forms are rare [6, 22, 32]. The study by Trope *et al.* [33] compared the number of roots and number of canals in mandibular premolars between African American and Caucasian patients. Their study reported results by number of patients rather than by the total number of teeth.

The African American group had an incidence of 2 or more roots in the mandibular second premolar tooth at 4.8% of the time compared with a 1.5% incidence in the Caucasian group. Although the incidence of multiple roots was greater in the African American patients compared with Caucasian patients in both the mandibular first and second premolars, the differences were statistically significant only for the mandibular first premolars. Ethnic differences in internal canal morphology were also found in this study but were not statistically significant. The African American group had an incidence of 2 or more canals in 7.8% of teeth, whereas the Caucasian group had an incidence of 2.8%.

***Access cavity preparation***

If in the diagnostic phase the presence of a third canal is suspected, the access cavity will present a mesiodistal extension in the buccal portion [33]. In three-rooted premolars, buccal roots lie close to each other and are often covered by a projection of cervical dentin.

**Table 3:** Incidence (%) of three canals in mandibular first premolars

**Author(s)**	**Year**	**Incidence (%)**
**Vertucci [** 17 **]**	1984	0.5
**Pineda and Kuttler [** 10 **]**	1972	0.9
**Caliskan ** ***et al. *** **[** 18 **]**	1995	5.7
**Zillich and Dawson [** 19 **]**	1973	0.4
**Yoshida ** ***et al. ***	2004	4.3
**Trope ** ***et al.*** ** [** 33 **]**	1986	African American 32.8 Caucasian 13.7

Consequently, the cavity will be T-shaped, because this is the only morphology that represents the coronal projection of the pulp chamber floor, allowing straight-line access to the canals [33]. 

***Instrumentation***

After completing the access cavity, similar to all other tooth types, the working length is determined. Thereafter, cleaning and shaping of the root canal is conducted. Firstly, the canals should be explored using small files (#8 and #10 for buccal canals and #15 for palatal or lingual canal) [6, 22, 31-33]. 

In mandibular premolars, usually the main canal orifice may split into two or three canals deep within the root. Thus, it is important to obtain straight line access to all the canals. This may be achieved by #4, 3 and 2 Gates-Glidden drills set on a slow handpiece rotating at 750 to 1000 rpm utilized in a crown down fashion. These drills should be withdrawn against the canal walls and away from the root concavities. This will reduce stress on the files used subsequently to shape the canals and minimize the risk of instrument separation and canal transportation [34, 35].

In maxillary premolars, hand instruments as well as various engine-driven instrumentation systems with various techniques such as crown-down pressure-less. It has been advised that primarily the radicular access should be achieved [33]. 

***Obturation***

Similar to other teeth, cold lateral compaction is the most common method for obturation of three-canalled premolars [21]. In maxillary premolars with three canals, in addition to the lateral compaction technique, hybrid technique has also been suggested to obturate the root canal system [33].

In hybrid technique the gutta-percha is thermoplasticized and compacted into root canal through motor-driven compactors. In mandibular premolars, due to the fact that the main canal orifice may split into two or three canals deep within the root, special considerations should be given to obturate the root canal system. According to Nallapati [23], after the master cones are selected and fit, a heat carrying plugger (Touch and Heat, SybronEndo, Orange, CA, USA) that binds 4 to 5 mm short of working length, is pre-fitted for each canal. The plugger is left in the canal to keep the canal patent while the adjacent canal is being obturated. 

The use of vertical compaction with apical backfilling technique has been shown to allow the creation of an effective apical plug and an excellent adaptation of backfilling to apical gutta-percha and to root canals. This technique includes seating the master cone and severing it with multiple heat bursts below the level of canal division zone. Backfilling of each canal to the level of furcation with the Obtura II (Obtura Corp, Fenton, MO) and compacting the warm gutta-percha with heated pluggers, complements the obturation process [21].

**Table 4:** Incidence (%) of three canals in mandibular second premolars

**Author(s)**	**Year**	**Incidence (%)**
**Zillich and Dawson**	1973	0.4
**Trope ** ***et al.***	1986	African American 7.8 Caucasian 2.8

## Conclusion

There are some clinical and radiographic parameters for diagnosing three-canalled premolars. Having a thorough understanding of normal anatomy, and of common variations, guarantees a successful treatment. The incidence of three root canals in maxillary first and second premolars varies from 0 to 6% and 0.3 to 2%, respectively. While for the mandibular first and second premolars this value varies from 0 to 33% and 0 to 8%, respectively. The access cavity is usually T-shaped and no specific instrumentation nor obturation method has been recommended for three-canalled premolars.
